# Anti-centromere antibody positivity is an independent variable associated with salivary gland ultrasonography score in Sjögren’s syndrome

**DOI:** 10.1038/s41598-024-55767-2

**Published:** 2024-03-04

**Authors:** Toshimasa Shimizu, Shin-ya Nishihata, Hideki Nakamura, Yukinori Takagi, Misa Sumi, Atsushi Kawakami

**Affiliations:** 1 Division of Advanced Preventive Medical Sciences, Department of Immunology and Rheumatology, Nagasaki University Graduate School of Biomedical Sciences, 1-7-1 Sakamoto, Nagasaki, 852-8501 Japan; 2 Clinical Research Center, Nagasaki University Hospital, Nagasaki, Japan; 3 Division of Hematology and Rheumatology, Department of Medicine, Nihon University School of Medicine, 30-1 Oyaguchi Kami-cho, Itabashi-ku, Tokyo, Japan; 4 Department of Radiology and Biomedical Informatics, Nagasaki University Graduate School of Biomedical Sciences, 1-7-1, Sakamoto, Nagasaki, 852-8588 Japan

**Keywords:** Sjögren’s syndrome, Salivary gland ultrasonography, Anti-centromere antibody, Focus score, Fibrosis, SjÃ¶gren's disease, Autoimmunity

## Abstract

Sjögren’s syndrome (SS) is an autoimmune disease characterized by periductal lymphocytic infiltration of the salivary and lacrimal glands. SS also exhibits extra-glandular manifestations and specific autoantibodies. Salivary gland ultrasonography (SGUS) is a common procedure used to assess the severity of glandular involvement. However, the association between SGUS and extra-glandular lesions remains poorly understood. This study aimed to identify clinical indices, including disease activity, associated with glandular involvement using SGUS in patients with SS. We included 115 patients with SS and 90 without SS. Patients with SS had significantly higher ultrasonography (US) score than patients without SS. Multivariate analysis revealed focus score, Saxon test positivity, and anti-centromere antibody (ACA) positivity as independent variables associated with the US score in patients with SS. In addition, these results were similar to those obtained in patients with primary SS. Patients with SS and ACA positivity had higher US score and an increased prevalence of hyperechoic bands in the parotid glands and submandibular glands. In conclusion, this study indicated that ACA positivity is associated with the US score in patients with SS. These results suggest that US findings in patients with ACA positivity might show specific changes in the salivary glands, especially fibrosis.

## Introduction

Sjögren’s syndrome (SS) is a systemic autoimmune disease characterized by periductal lymphocytic infiltration of the salivary and lacrimal glands, resulting in reduced secretory function and oral and ocular dryness. Additionally, patients with SS often present with extra-glandular lesions, including interstitial lung disease and interstitial nephritis, accompanied by autoantibodies such as anti-Ro/SS-A and La/SS-B antibodies^1^.

Salivary gland involvement in SS has been traditionally evaluated using imaging techniques such as sialography, scintigraphy, and magnetic resonance imaging^2^. However, salivary gland ultrasonography (SGUS) has emerged as a useful tool in evaluating salivary gland involvement^3–5^. SGUS offers the advantage of noninvasive, real-time evaluation of structural changes and sialadenitis in the major salivary glands, namely the submandibular and parotid glands, enabling the assessment of salivary gland disorders.

Assessing glandular lesions is essential for confirming xerostomia and determining oral hygiene status. Additionally, the severity of glandular lesions in SS can reflect the severity and prognosis of the systemic condition, as studies have indicated an association between the intensity of lymphocytic infiltration in the salivary glands and systemic activity, such as the European League Against Rheumatism (EULAR) SS Disease Activity Index (ESSDAI)^6^. Recent reports have examined the association between salivary gland lesions evaluated using SGUS and clinical characteristics, including systemic activity and autoantibody levels^7–13^. However, the findings from these studies have been inconsistent, with some showing an association between systemic activity and SGUS, whereas others have not, which is room for consideration.

Thus, this study aimed to identify the clinical indices, including autoantibodies and disease activity, associated with glandular involvement using SGUS in patients with SS.

## Materials and methods

### Patients

We consecutively enrolled patients with suspected SS who visited our clinic and underwent labial salivary gland biopsy and SGUS between April 1995 and March 2020. Patients with SS were classified based on the 2002 American–European Consensus Group (AECG) SS classification criteria^14^. To compensate for the lack of data on unstimulated whole-saliva flow in many cases, we substituted the Saxon test result of ≤ 2 g/2 min to satisfy the low salivary volume requirement in the AECG SS classification criteria. The patients without SS exhibited sicca symptoms but did not fulfill the AECG SS classification criteria. In addition, we classified primary SS (pSS) based on the 2016 American College of Rheumatology (ACR)/EULAR pSS classification criteria^15^. We also substituted the Saxon test result of ≤ 2 g/2 min to satisfy the low salivary volume requirement criteria of the 2016 ACR/EULAR classification to compensate for the lack of data on unstimulated whole-saliva flow in many cases. We excluded patients who had undergone SGUS evaluation more than 1 year after the SS diagnosis. Various clinical indices, such as patient age, sex, sicca symptoms, Saxon test results, Schirmer test results, anti-Ro/SS-A antibody positivity, anti-La/SS-B antibody positivity, anti-centromere antibody (ACA) positivity, rheumatoid factor (RF) positivity, serum immunoglobulin G (IgG) levels, ESSDAI, and clinical ESSDAI (ClinESSDAI) scores, were obtained from medical records^16,17^. Anti-Ro/SS-A antibody, anti-La/SS-B antibody, and ACA were measured by an enzyme-linked immunosorbent assay (ELISA) (anti-Ro/SS-A: Mesacup SS-A/Ro test or Mesacup-2 SS-A/Ro test, anti-La/SS-B: Mesacup SS-B/La test, Mesacup-2 SS-B/La test or Mesacup-3 SS-B/La test, ACA: Mesacup CENP-B test or Mesacup-2 CENP-B test; Medical & Biological Laboratories, Nagoya, Japan). Standard values were defined per the product manual. Calculation of the focus score (FS) of the labial salivary glands (LSGs) followed the standardization method endorsed by the EULAR SS Study Group^18^. To determine the FS, which represents the number of foci per 4 mm^2^ in the LSGs, the number of foci in a section from the LSGs was counted, and the surface area of the section was measured using a hybrid cell count system mounted on a microscope (BZ-X700; Keyence, Osaka, Japan).

The study was conducted in accordance with the Declaration of Helsinki and Ethical Guidelines for Life Sciences and Medical Research Involving Human Subjects. Nagasaki University Hospital Clinical Research Ethics Committee approved this study (Approval no. 20091428). The ethics committee has excused the necessity of obtaining a patient's written informed consent according to the local regulations for a retrospective observational study.

### Ultrasonographic (US) examinations and measurement of US score

**Figure 1:**
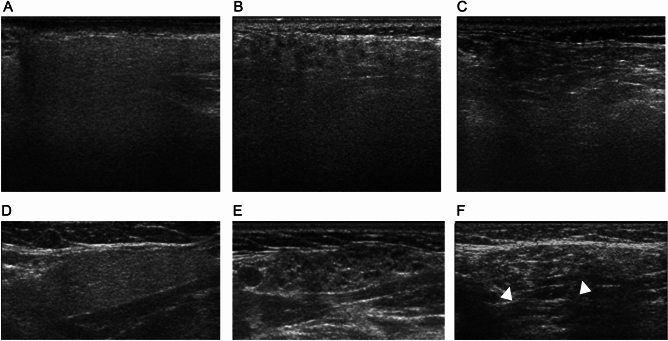
Representative ultrasonography findings of parotid and submandibular glands in patients with Sjögren's syndrome and those without Sjögren's syndrome. (**A** and **D**) Patients without Sjögren's syndrome. Ultrasonography shows hypoechoic areas (–) and hyperechoic bands (–) in parotid (**A**) and submandibular (**D**) glands. (**B**,**C**,**E**,**F**) Patients with Sjögren's syndrome. Hypoechoic areas (+) in parotid (**B**) and submandibular (**E**) glands. Hyperechoic bands (+) in parotid (**C**). Hyperechoic bands (+) and irregular margins (+) (arrowheads) in submandibular (**F**) glands.

Gray-scale US of the parotid and submandibular glands was performed at 14 MHz using LOGIQ 9 (2003 and later) or LOQIG 700 (1995–2002) equipment with a wide bandwidth (9–14 MHz) (GE Healthcare, Milwaukee, WI, USA). The US score was defined based on previously reported criteria, involving cumulative scores (1 score per positive finding) for hypoechoic areas and hyperechoic bands in parotid glands (PGs) and submandibular glands (SMGs), as well as irregular margins of both SMGs, distributing from 0 point (normal) to 10 point (maximum) (Fig. 1)^5^. We did not use the recent US score proposed by the Outcome Measures in Rheumatology Clinical Trials (OMERACT) Working Group with a four-grade semiquantitative and two-item qualitative scoring system^19^. To examine the association between US findings and clinical indices in more detail, we used our proposed scoring system, which allows the scoring of US findings to for each finding within each salivary gland, rather than the OMERACT system. The number of years of SGUS experience is crucial for consistency in the SGUS evaluations^20^. Two radiologists with 23 years of experience in using US to diagnose salivary glands in patients with SS evaluated the US images obtained from the transverse planes of the PGs and SMGs while being blinded to patients’ clinical information. Both radiologists finally agreed on the conclusion.

### Statistical analysis

Differences between the two groups were determined using Fisher’s exact test for discrete variables and the Mann–Whitney U test for continuous variables. The correlation between FS and US score was determined using Spearman’s rank correlation test. Subsequently, we explored the variables associated with the US score in patients with SS using analysis of covariance. Multivariate analysis was performed using variables that were identified as relevant in the univariate analysis.

Our dataset contained missing data for certain clinical indices, with a proportion of missing values of 5.7%. To enhance statistical power and mitigate selection bias within the models, we performed multivariate imputation by chained equations (MICE) method^21^. Each imputation model included the following variables: US score, FS, age, sex, sicca symptoms, Saxon test results, Schirmer test results, anti-Ro/SS-A antibody positivity, anti-La/SS-B antibody positivity, ACA positivity, serum IgG levels, ESSDAI, and ClinESSDAI scores. We generated 200 imputations and pooled the coefficient estimates using Rubin’s rules.

Statistical analyses were performed using the R studio software (version 4.3.1), especially the MICE package (version 3.16.0) for multiple imputations, JMP Pro software (version 17.0; SAS Institute Inc., Cary, NC), and GraphPad Prism (version 9.51; GraphPad Software, La Jolla, CA). Statistical significance was set at p < 0.05.

## Results

### Patient characteristics

Table 1 summarizes the characteristics of 115 patients with SS who fulfilled the AECG SS classification criteria and 90 patients without SS who fulfilled the AECG SS classification criteria. The SS group had a significantly higher proportion of female patients, LSG biopsy FS, prevalence of xerophthalmia, Saxon test positivity, Schirmer’s test positivity, anti-SS-A/Ro antibody positivity, anti-SS-B/La antibody positivity, and serum IgG levels than the non-SS sicca group. Among the 115 patients with SS, 96 were classified as having primary SS, whereas 19 were classified as having secondary SS. The distribution of autoimmune diseases accompanying secondary SS included rheumatoid arthritis, systemic lupus erythematosus, rheumatoid arthritis with systemic lupus erythematosus, rheumatoid arthritis with mixed connective tissue disease, and idiopathic inflammatory myopathy (n = 11, 5, 1, 1, and 1, respectively). In addition, 106 patients with pSS fulfilled the 2016 ACR/EULAR pSS classification criteria. Supplementary Table S1 summarizes the characteristics of 106 patients with pSS who fulfilled the 2016 ACR/EULAR pSS classification criteria.

**Table 1:** Demographic and clinical characteristics of patients with Sjögren’s syndrome and patients without Sjögren’s syndrome.

Variables	SS (n = 115)	Non-SS (n = 90)	*p-value*
Age (years), median with IQR	61 (52–69)	61 (52–70)	0.94^c^
Female, n (%)	109 (94.8)	78 (86.7)	**0.049**^d^
LSG biopsy, focus score, median with IQR	2.57 (1.31–4.8)	0 (0–0.93)	** < 0.001**^c^
Xerostomia, n (%)	88 (77.9) (n = 113)	63 (70.8) (n = 89)	0.26^d^
Xerophthalmia, n (%)	76 (67.3) (n = 113)	33 (37.1) (n = 89)	** < 0.001**^d^
Saxon test positivity, n (%)	87 (80.6) (n = 108)	44 (55.7) (n = 79)	** < 0.001**^d^
Schirmer’s test positivity, n (%)	75 (70.1) (n = 107)	36 (45) (n = 80)	** < 0.001**^d^
Anti-Ro/SS-A antibody positivity, n (%)	88 (76.5)	19 (21.1)	** < 0.001**^d^
Anti-La/SS-B antibody positivity, n (%)	32 (28.6) (n = 112)	3 (3.4) (n = 88)	** < 0.001**^d^
Anti-centromere antibody positivity, n (%)	26 (23) (n = 113)^a^	13 (14.8) (n = 88)^b^	0.14^d^
RF positivity, n (%)	48 (52.2) (n = 92)	37 (45.1) (n = 82)	0.37^d^
Serum IgG ≥ 1600 mg/dL, n (%)	61 (55.5) (n = 110)	28 (34.6) (n = 81)	**0.0053**^d^
ESSDAI score, median with IQR	2 (0–6) (n = 87)	NA	NA
ClinESSDAI score, median with IQR	2 (0–6) (n = 87)	NA	NA

SS, Sjögren's syndrome; LSG, labial salivary gland; RF, rheumatoid factor; IgG, immunoglobulin G; ESSDAI, European League Against Rheumatism Sjögren's Syndrome Disease Activity Index; IQR, interquartile range; NA, not assessed.

^a^Anti-centromere antibody with anti-Ro/SS-A antibody: n = 12; anti-centromere antibody without anti-Ro/SS-A antibody (n = 14).

^b^All participants had anti-centromere antibody without anti-Ro/SS-A antibody.

^c^Mann–Whitney U test.

^d^Fisher's exact test.* p* < 0.05 was considered significant. Bold font indicates significant values.

### Comparison of US score between the SS and non-SS sicca groups

**Figure 2:**
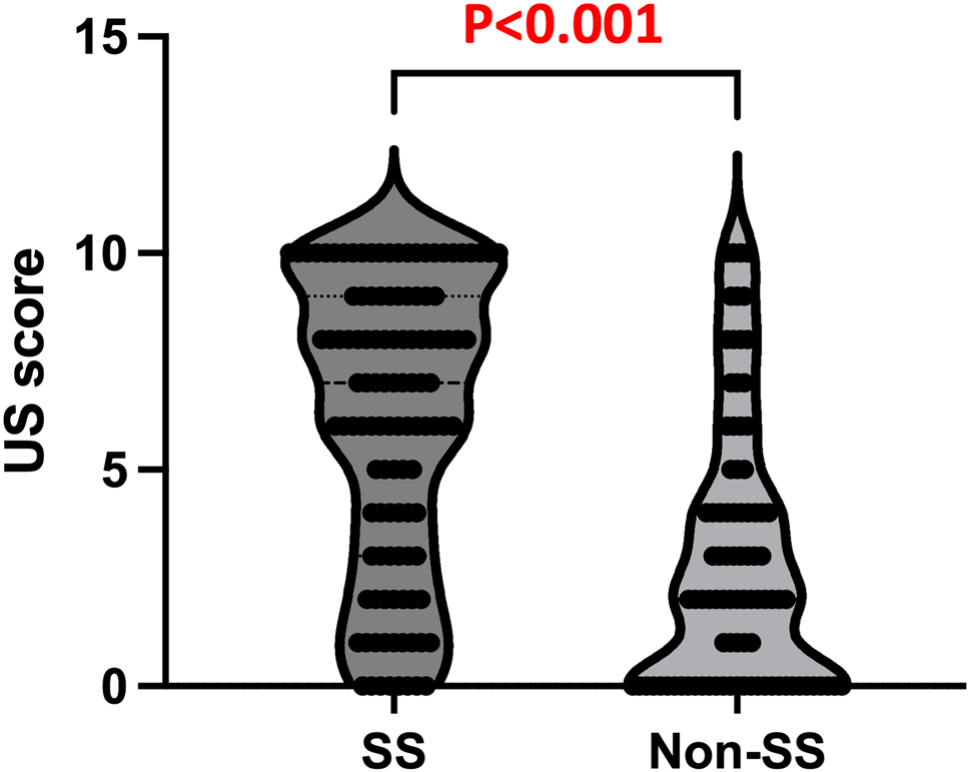
Salivary gland ultrasonography score in patients with Sjögren’s syndrome and those without Sjögren’s syndrome. The salivary gland ultrasonography score between patients with Sjögren’s syndrome (n = 115) and those without Sjögren’s syndrome (n = 90) were compared using the Mann–Whitney U test. The distributions of the ultrasonography score are presented using violin plots, which included all data points. Statistical significance was set at P < 0.05. SS, Sjögren's syndrome; US, ultrasonography.

**Figure 3:**
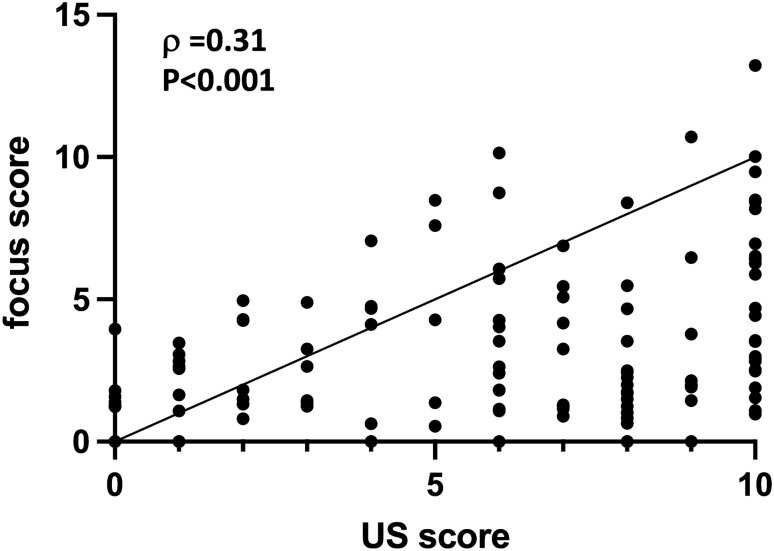
Correlation between ultrasonography score and focus score in patients with Sjögren’s syndrome. Correlation analysis was performed using Spearman's rank correlation coefficient. Statistical significance was set at P < 0.05. N = 115. US, ultrasonography.

Patients with SS had significantly higher US score than patients without SS (Fig. 2). Additionally, a significant positive correlation between US score and FS was observed in patients with SS (Fig. 3).

### Variables associated with US score in patients with SS

We performed an analysis of covariance to explore the variables associated with the US score in patients with SS. Univariate analysis identified FS, Saxon test positivity, ACA positivity, and high serum IgG levels as variables associated with the US score in patients with SS with complete cases (Table 2). Additionally, multivariate analysis revealed that FS, Saxon test positivity, and ACA positivity were independent variables associated with US score in patients with SS with complete cases (Table 2). These results were similar to those obtained in the pSS group (Supplementary Table S2). Table 3 presents the results of the analysis of covariance for variables associated with the US score in patients with SS using a multiple imputation model. Consistent with the complete-case analysis, ACA positivity was identified as an independent variable associated with the US score (Table 3). Additionally, in patients with pSS according to the 2016 ACR/EULAR pSS classification criteria, ACA positivity in addition to FS, Saxon test positivity, and anti-La/SS-B antibody positivity were independent variables associated with US score (Supplementary Table S3).

**Table 2:** Variables associated with US score in patients with Sjögren’s syndrome from analysis of covariance with complete cases.

Variables	Unadjusted			Adjusted*		
	Coefficient	95% CI	*p-value*	Coefficient	95% CI	*p-value*
Age, per 1 year (n = 115)	0.001	− 0.04 to 0.04	0.96			
Female (n = 115)	− 0.06	− 2.8 to 2.69	0.97			
Focus score, per 1 (n = 115)	0.21	0.07 to 0.36	**0.0035**	0.27	0.06 to 0.49	**0.015**
Xerostomia (+) (n = 113)	0.5	− 0.98 to 1.99	0.51			
Xerophthalmia (+) (n = 113)	0.23	− 1.09 to 1.54	0.74			
Saxon test (+) (n = 108)	2.86	1.36 to 4.37	** < 0.001**	2.51	1.02 to 3.99	**0.0013**
Schirmer’s test (+) (n = 107)	0.34	− 1.04 to 1.72	0.63			
Anti-Ro/SS-A antibody (+) (n = 115)	− 0.14	− 1.58 to 1.3	0.85			
Anti-La/SS-B antibody (+) (n = 112)	1.26	− 0.08 to 2.6	0.067			
Anti-centromere antibody (+) (n = 113)	2.37	0.98 to 3.77	**0.0012**	1.73	0.31 to 3.16	**0.019**
Serum IgG ≥ 1600 mg/dL (+) (n = 110)	1.33	0.1 to 2.57	**0.037**	1	− 0.18 to 2.17	0.099
ClinESSDAI, per 1 (n = 87)	0.12	− 0.01 to 0.25	0.078			
ESSDAI, per 1 (n = 87)	0.14	− 0.01 to 0.28	0.063			

SS, Sjögren's syndrome; CI, confidence interval; IgG, immunoglobulin G; ESSDAI, European League Against Rheumatism; Sjögren's Syndrome Disease Activity Index. *N = 104 for the multivariate analysis. *p* < 0.05 was considered significant. Bold font indicates significant values.

**Table 3:** Variables associated with US score in patients with Sjögren’s syndrome from analysis of covariance with multiple imputations.

Variables	Unadjusted			Adjusted		
	Coefficient	95% CI	*p-value*	Coefficient	95% CI	*p-value*
Age, per 1 year	0.001	− 0.04 to 0.04	0.96			
Female	− 0.06	− 2.83 to 2.72	0.97			
Focus score, per 1	0.21	0.07 to 0.36	**0.0035**	0.17	0.03 to 0.31	**0.016**
Xerostomia (+)	0.6	− 1.04 to 1.94	0.55			
Xerophthalmia (+)	0.24	− 1.08 to 1.56	0.72			
Saxon test (+)	2.64	1.1 to 4.18	** < 0.001**	2.47	1.05 to 3.89	** < 0.001**
Schirmer’s test (+)	0.26	− 1.13 to 1.64	0.71			
Anti-Ro/SS-A antibody (+)	− 0.14	− 1.6 to 1.31	0.85			
Anti-La/SS-B antibody (+)	1.29	− 0.08 to 2.65	0.064			
Anti-centromere antibody (+)	2.42	1.03 to 3.81	** < 0.001**	2.05	0.73 to 3.36	**0.0026**
Serum IgG ≥ 1600 mg/dl (+)	1.26	0.03 to 2.5	**0.045**	1.15	0.02 to 2.27	**0.045**
ClinESSDAI, per 1	0.08	− 0.06 to 0.22	0.25			
ESSDAI, per 1	0.1	− 0.05 to 0.25	0.20			

SS, Sjögren's syndrome; CI, confidence interval; IgG, immunoglobulin G; ESSDAI, European League Against Rheumatism; Sjögren's Syndrome Disease Activity Index.

*p* < 0.05 was considered significant. Bold font indicates significant values.

### US score and FS in SS patients with ACA and without ACA

**Figure 4:**
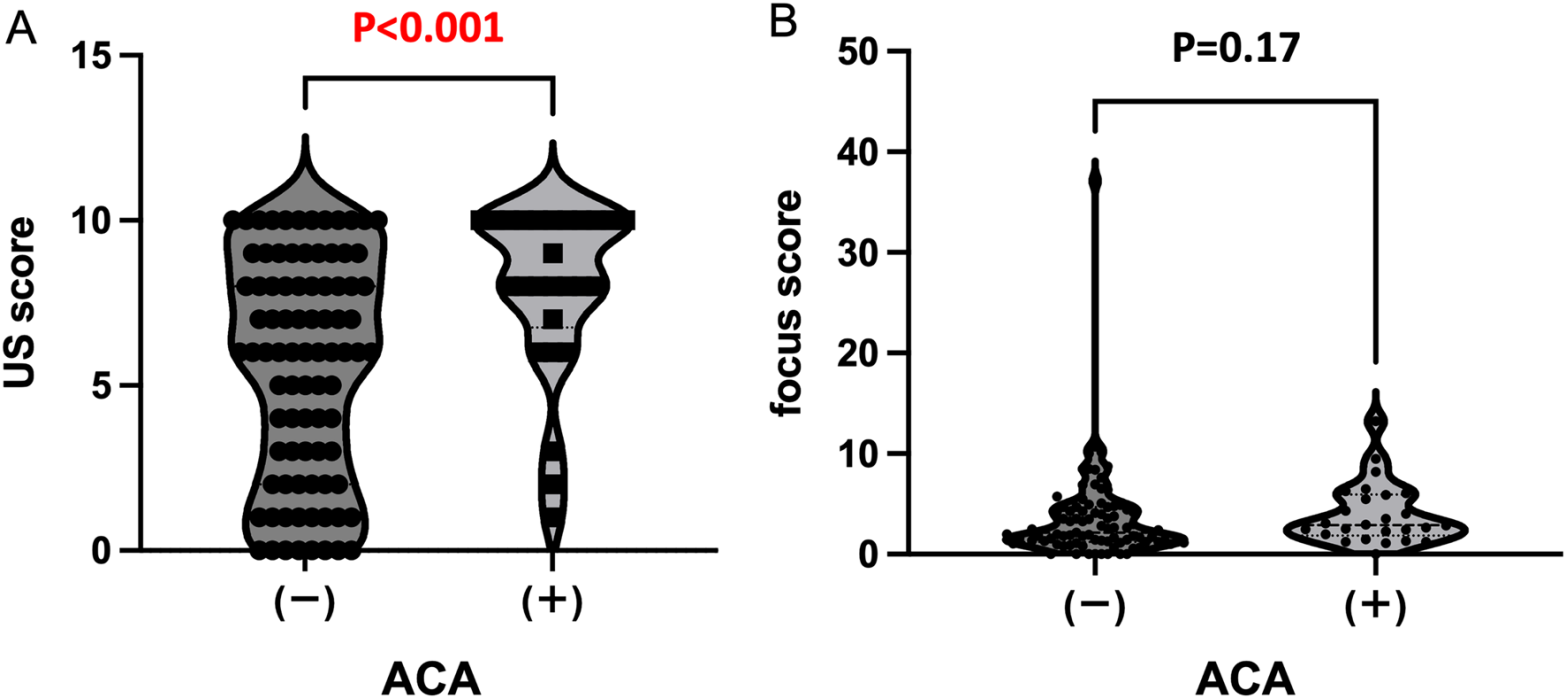
Ultrasonography score and focus score in patients with Sjögren's syndrome and anti-centromere antibody and those without anti-centromere antibody. The ultrasonography score (**A**) and focus score (**B**) between patients with Sjögren's syndrome and anti-centromere antibody (n = 26) and those without anti-centromere antibody (n = 87) were compared using the Mann–Whitney U test. The distributions are presented using violin plots, which included all the data points. Statistical significance was set at P < 0.05. ACA, anti-centromere antibody; SS, Sjögren's syndrome; US, ultrasonography.

As ACA positivity was identified as one of the variables independently associated with the US score in patients with SS, we compared the US score and FS between patients with SS with and without ACA (Fig. 4).

**Figure 5:**
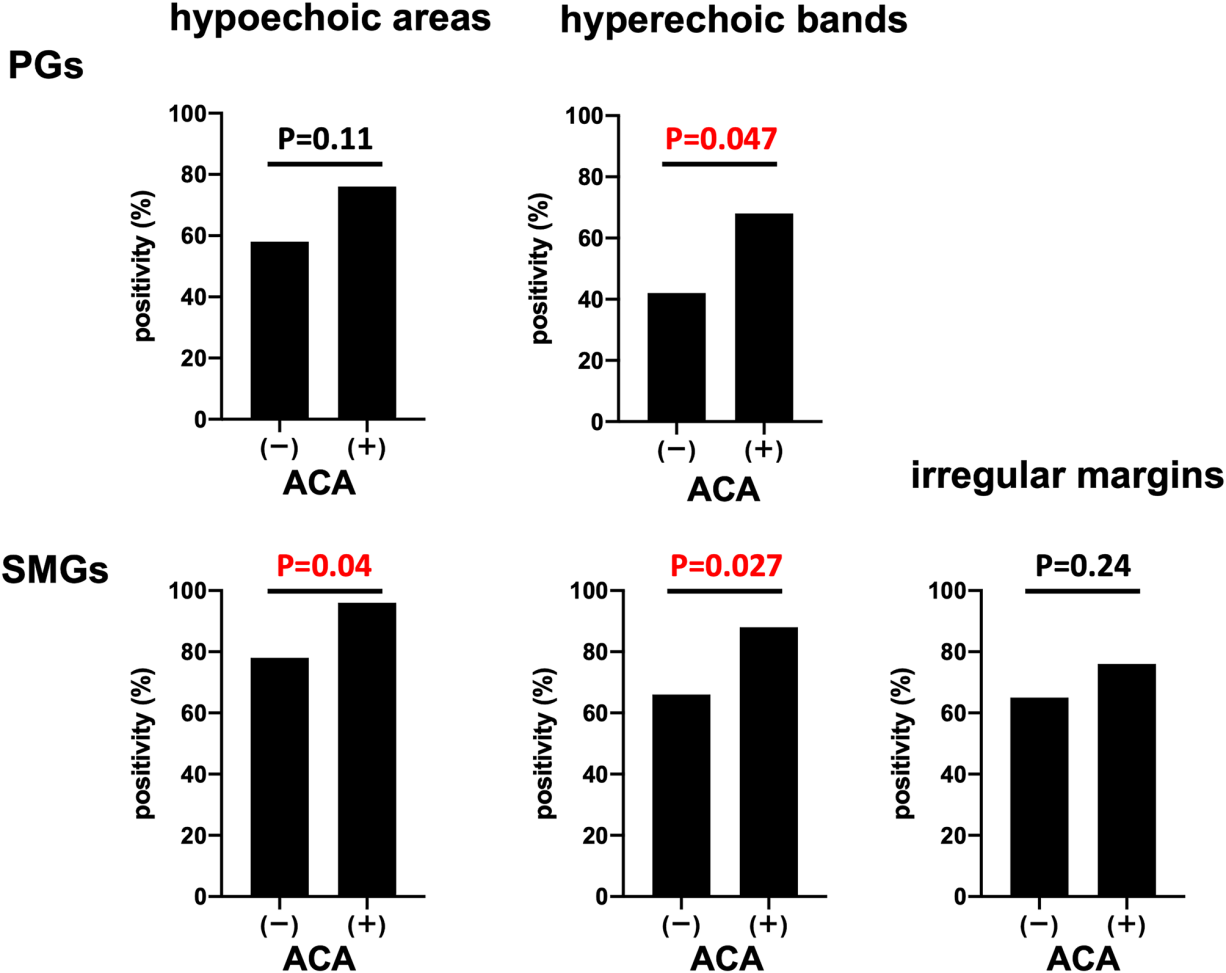
Ultrasonography findings in patients with Sjögren's syndrome and anti-centromere antibody and those without anti-centromere antibody. The proportion of each ultrasonographic finding (hypoechoic areas and hyperechoic bands in the bilateral parotid glands and submandibular glands, and irregular margins of bilateral submandibular glands) between patients with Sjögren's syndrome and anti-centromere antibody (n = 26) and those without anti-centromere antibody (n = 87) were compared using Fisher’s exact test. ACA, anti-centromere antibody; PGs, parotid gland; SMGs, submandibular gland. Statistical significance was set at P < 0.05.

Patients with SS and ACA exhibited significantly higher US score than those without ACA (Fig. 4A). However, the FS did not differ significantly between patients with and without ACA (Fig. 4B). Furthermore, patients with ACA showed a significantly higher presence of hypoechoic areas in the SMGs and hyperechoic bands in the PGs and SMGs than those without ACA (Fig. 5). In the patients with SS with ACA, twelve patients were positive for anti-Ro/SS-A antibody, and fourteen patients were negative for anti-Ro/SS-A antibody. There were no differences in the US score and FS between ACA-positive patients with and without anti-Ro/SS-A antibodies (Supplementary Fig. S1).

## Discussion

This study investigated the clinical indices associated with glandular involvement using SGUS in patients with SS. The results revealed that the US score was positively correlated with FS in patients with SS. Furthermore, one of the independent variables associated with US scores in patients with SS was the presence of ACA.

SGUS has gained recognition as a valuable tool for diagnosing and monitoring treatment efficacy in SS^4,5,22,23^, increasing the demand for its use as a diagnostic and activity assessment tool for SS. Our findings revealed that patients with SS had higher US score than patients without SS who exhibited xerostomia symptoms but did not meet the classification criteria for SS. Furthermore, a positive correlation was observed between the FS and US score in patients with SS, indicating a potential reflection of sialadenitis.

Moreover, the recognition of the significance of the link between glandular and extra-glandular lesions that spread to the systemic organs in SS is increasing. Actually, several studies have explored the association between SGUS and clinical features, including systemic activity and serology. Previous reports have revealed that patients with abnormal SGUS findings have a higher ESSDAI, elevated FS, and reduced salivary flow^7,8,10,11^. In contrast, there have been reports of no association between SGUS abnormalities and ESSDAI^9,13^. We observed a trend toward an association between the US score and ESSDAI; however, the difference was insignificant. Regarding immunological findings, previous studies have demonstrated patients with abnormal SGUS findings, including higher US score, had more frequent anti-Ro/SS-A antibody, anti-La/SS-B antibody, RF positivity, and hypergammaglobulinemia^7–12^. In our study, factors associated with the US score include the FS and Saxon test results, which directly reflect inflammation and salivary gland disorders, and the presence of ACA rather than anti-Ro/SS-A and anti-La/SS-B antibodies.

ACA is a specific autoantibody observed in a subset of patients with limited cutaneous systemic sclerosis^24^. However, it has also been detected in 3.7–27% of patients with SS^25^. ACA has also been reported in patients with cancers and autoimmune diseases other than systemic sclerosis and SS^26–28^. In this study, patients with ACA positivity did not exhibit findings suggestive of systemic sclerosis, such as thickened or hardened skin. In addition, there were no differences in autoimmune disease and cancer complications between patients with and without ACA (data not shown). Patients with SS and ACA exhibited distinct clinical characteristics than those without ACA. These characteristics include an older age at disease onset; a higher incidence of Raynaud's phenomenon, peripheral neuropathy, gastroesophageal involvement, and pulmonary involvement; a lower prevalence of anti-Ro/SS-A and anti-La/SS-B antibodies and rheumatoid factor; and a reduced frequency of leukocytopenia and hypergammaglobulinemia^24,29–31^. In addition, several studies have shown differences in clinical characteristics among ACA-positive SS with and without anti-Ro/SS-A antibodies, indicating that anti-Ro/SS-A positive cases have higher serum IgG levels and ESSDAI than anti-Ro/SS-A negative cases^32,33^. One study found no difference in FS between ACA-positive patients with and without anti-Ro/SS-A antibodies^33^; however, differences in the salivary gland disorders between the two groups have not been fully established. In this study, the US score and FS did not differ between ACA-positive patients with and without anti-Ro/SS-A antibodies. However, this study had a small sample size, and further studies are needed to verify our results.

Our study observed that patients with ACA positivity had higher US score than patients with ACA negativity, especially showing a higher percentage of hyperechoic bands, whereas the FS did not differ between the two groups.

The histopathological findings corresponding to abnormal SGUS findings are not yet fully understood. However, several reports have been published regarding these findings. The hypoechoic areas reflect inflammatory cell infiltration, whereas hyperechoic bands may indicate glandular damage and dense fibrous tissue deposition^34–37^. Regarding histopathological findings in patients with SS and ACA, no difference was observed in lymphocytic infiltration of the minor salivary glands between patients with SS with and without ACA^38^. However, fibrous tissue stained blue via Azan Mallory staining was more severe in the minor salivary glands of patients with SS and ACA than in those without ACA^39^. Based on these findings, higher US score, particularly an increased percentage of hyperechoic bands in patients with ACA, may reflect changes in the salivary gland, such as fibrosis. However, one study reported contrasting findings, wherein patients with SS and ACA had lower US score than those without ACA, and no difference was observed in the extent of hyperechoic foci between SS patients with and without ACA^40^. In the previous study, US findings were scored based on a study by Hocevar et al., who evaluated five US parameters (including echogenicity, inhomogeneity, number of hypoechogenic areas, hyperechogenic reflections, and clearness of the borders of the salivary gland)^41^. Despite variations in the definition of the US score, including number and weight of scoring items, further investigations are required to elucidate the underlying pathogenesis linking ACA and US findings.

Our study had some limitations. First, it had a retrospective observational design in which the patients’ backgrounds could not be adjusted because of the small number of patients. Second, regarding the classification of SS, the Saxon test was used instead of unstimulated whole-saliva flow to evaluate low salivary volume; thus, some cases could not be classified as SS without a positive Saxon test. Additionally, some participants without SS did not perform the items in the classification criteria, and if these items had been performed, they might have included cases classified as SS. Third, the dataset contained missing values for some variables that could not be analyzed for all participants. Nevertheless, we conducted an additional investigation employing multiple imputations to complement the missing values to address this limitation. The outcomes were similar, reinforcing the robustness of our findings. Finally, this study did not analyze cases using the US score recently proposed by the OMERACT working group^19^. We believe that our proposed scoring system provides a more detailed assessment of salivary gland lesions due to its incorporation of a greater number of scoring items compared to the OMERACT system. However, further studies are required to examine the difference in usefulness between the OMERACT system and our scoring system.

## Conclusion

This study revealed that ACA positivity, besides FS and Saxon test, which reflect salivary gland disorders in SS, is associated with US score in patients with SS. These findings imply that US findings in patients with ACA positivity may exhibit specific changes in the salivary glands, such as fibrosis and sialadenitis. These findings may help elucidate SS pathogenesis; however, more detailed studies are warranted.

### Supplementary Information

Supplementary Information.

## Data Availability

The data that support the findings of this study are available from the corresponding author upon reasonable request.
