# Supplementary material for: Anti-centromere antibody positivity is an independent variable associated with salivary gland ultrasonography score in Sjögren’s syndrome
Source: Sci Rep. 2024 Mar 4;14:5303. doi: 10.1038/s41598-024-55767-2 (PMC10912294; doi:10.1038/s41598-024-55767-2)
Supplement: Supplementary file 1 — Supplementary Information. [file 41598_2024_55767_MOESM1_ESM.docx]

**Supplementary Table S1. Demographic and clinical characteristics of patients with primary Sjögren’s syndrome who fulfilled the 2016 American College of Rheumatology/European League Against Rheumatism primary Sjögren’s syndrome classification criteria**

| Variables | SS (n = 106) |
| --- | --- |
| Age (years), median with IQR | 61 (50–70) |
| Female, n (%) | 99 (93.4) |
| LSG biopsy, focus score, median with IQR | 2.51 (1.26–4.67) |
| Xerostomia, n (%) | 80 (76.9) (n = 104) |
| Xerophthalmia, n (%) | 70 (67.3) (n = 104) |
| Saxon test positivity, n (%) | 80 (79.2) (n = 101) |
| Schirmer’s test positivity, n (%) | 66 (66.7) (n = 99) |
| Anti-Ro/SS-A antibody positivity, n (%) | 78 (73.6) |
| Anti-La/SS-B antibody positivity, n (%) | 31 (29.8) (n = 104) |
| Anti-centromere antibody positivity, n (%) | 26 (24.8) (n = 105)^a^ |
| RF positivity, n (%) | 41 (48.2) (n = 85) |
| Serum IgG ≥ 1600 mg/dL, n (%) | 59 (57.8) (n = 102) |
| ESSDAI score, median with IQR | 3 (0­–6) (n = 73) |
| ClinESSDAI score, median with IQR | 2 (0­–5.5) (n = 73) |

SS, Sjögren's syndrome; LSG, labial salivary gland; RF, rheumatoid factor; IgG, immunoglobulin G; ESSDAI, European League Against Rheumatism Sjögren's Syndrome Disease Activity Index; IQR, interquartile range; NA, not assessed. ^a^Anti-centromere antibody with anti-Ro/SS-A antibody: n = 11; anti-centromere antibody without anti-Ro/SS-A antibody: n = 15.

**Supplementary Table S2. Variables associated with US score in patients with primary Sjögren’s syndrome who fulfilled the American–European Consensus Group Sjögren’s syndrome classification criteria from the analysis of covariance with complete cases**

| Variables | Unadjusted | | | Adjusted^*^ | | |
| --- | --- | --- | --- | --- | --- | --- |
|  | Coefficient | 95% CI | *p-value* | Coefficient | 95% CI | *p-value* |
| Age, per 1 year  (n = 96) | 0.01 | –0.04 to 0.06 | 0.66 |  |  |  |
| Female  (n = 96) | 0.64 | –2.43 to 3.71 | 0.68 |  |  |  |
| Focus score, per 1  (n = 96) | 0.21 | 0.06 to 0.36 | **0.0060** | 0.25 | 0.01 to 0.48 | **0.043** |
| Xerostomia (+)  (n = 94) | 0.46 | –1.24 to 2.15 | 0.60 |  |  |  |
| Xerophthalmia (+)  (n = 94) | 0.17 | –1.38 to 1.72 | 0.83 |  |  |  |
| Saxon test (+)  (n = 91) | 3.2 | 1.59 to 4.8 | **<0.001** | 2.86 | 1.26 to 4.47 | **<0.001** |
| Schirmer’s test (+)  (n = 90) | 0.63 | –0.9 to 2.17 | 0.42 |  |  |  |
| Anti-Ro/SS-A antibody (+)  (n = 96) | 0.06 | –1.52 to 1.63 | 0.95 |  |  |  |
| Anti-La/SS-B antibody (+)  (n = 94) | 1.38 | –0.05 to 2.82 | 0.062 |  |  |  |
| Anti-centromere antibody (+)  (n = 95) | 2.32 | 0.84 to 3.81 | **0.0028** | 1.59 | 0.1 to 3.07 | **0.039** |
| Serum IgG≧1600 mg/dL (+)  (n = 92) | 1.74 | 0.37 to 3.1 | **0.014** | 1.27 | -0.04 to 2.57 | 0.060 |
| ClinESSDAI, per 1  (n = 71) | 0.13 | –0.04 to 0.3 | 0.13 |  |  |  |
| ESSDAI, per 1  (n = 71) | 0.15 | –0.03 to 0.34 | 0.10 |  |  |  |

SS, Sjögren's syndrome; CI, confidence interval; IgG, immunoglobulin G; ESSDAI, European League Against Rheumatism; Sjögren's Syndrome Disease Activity Index. ^*^N = 87 for the multivariate analysis. *p* < 0.05 was considered significant. Bold font indicates significant values.

**Supplementary Table S3. Variables associated with US score in patients with primary Sjögren’s syndrome who fulfilled the 2016 American College of Rheumatology/European League Against Rheumatism primary Sjögren’s syndrome classification criteria from analysis of covariance with complete cases**

| Variables | Unadjusted | | | Adjusted^*^ | | |
| --- | --- | --- | --- | --- | --- | --- |
|  | Coefficient | 95% CI | *p-value* | Coefficient | 95% CI | *p-value* |
| Age, per 1 year  (n = 106) | 0.03 | –0.02 to 0.07 | 0.27 |  |  |  |
| Female  (n = 106) | 1.38 | –1.34 to 4.1 | 0.32 |  |  |  |
| Focus score, per 1  (n = 106) | 0.26 | 0.11 to 0.41 | **<0.001** | 0.36 | 0.15 to 0.58 | **0.0012** |
| Xerostomia (+) (n = 104) | 0.73 | –0.88 to 2.33 | 0.38 |  |  |  |
| Xerophthalmia (+)  (n = 104) | 0.89 | –0.54 to 2.33 | 0.23 |  |  |  |
| Saxon test (+) (n = 101) | 2.69 | 1.08 to 4.3 | **0.0015** | 2.06 | 0.57 to 3.55 | **0.008** |
| Schirmer’s test (+)  (n = 99) | 0.91 | –0.55 to 2.37 | 0.23 |  |  |  |
| Anti-Ro/SS-A antibody (+)  (n = 106) | 0.24 | –1.27 to 1.76 | 0.75 |  |  |  |
| Anti-La/SS-B antibody (+)  (n = 104) | 1.83 | 0.4 to 3.26 | **0.014** | 1.95 | 0.66 to 3.23 | **0.0038** |
| Anti-centromere antibody (+)  (n = 105) | 2.8 | 1.34 to 4.26 | **<0.001** | 2.31 | 0.9 to 3.72 | **0.0018** |
| Serum IgG≧1600 mg/dL (+)  (n = 102) | 1.1 | –0.28 to 2.47 | 0.12 |  |  |  |
| ClinESSDAI, per 1(n = 73) | 0.06 | –0.1 to 0.23 | 0.47 |  |  |  |
| ESSDAI, per 1 (n = 73) | 0.1 | –0.08 to 0.28 | 0.29 |  |  |  |

SS, Sjögren's syndrome; CI, confidence interval; IgG, immunoglobulin G; ESSDAI, European League Against Rheumatism; Sjögren's Syndrome Disease Activity Index. ^*^N = 98 for the multivariate analysis. *p* < 0.05 was considered significant. Bold font indicates significant values.

**
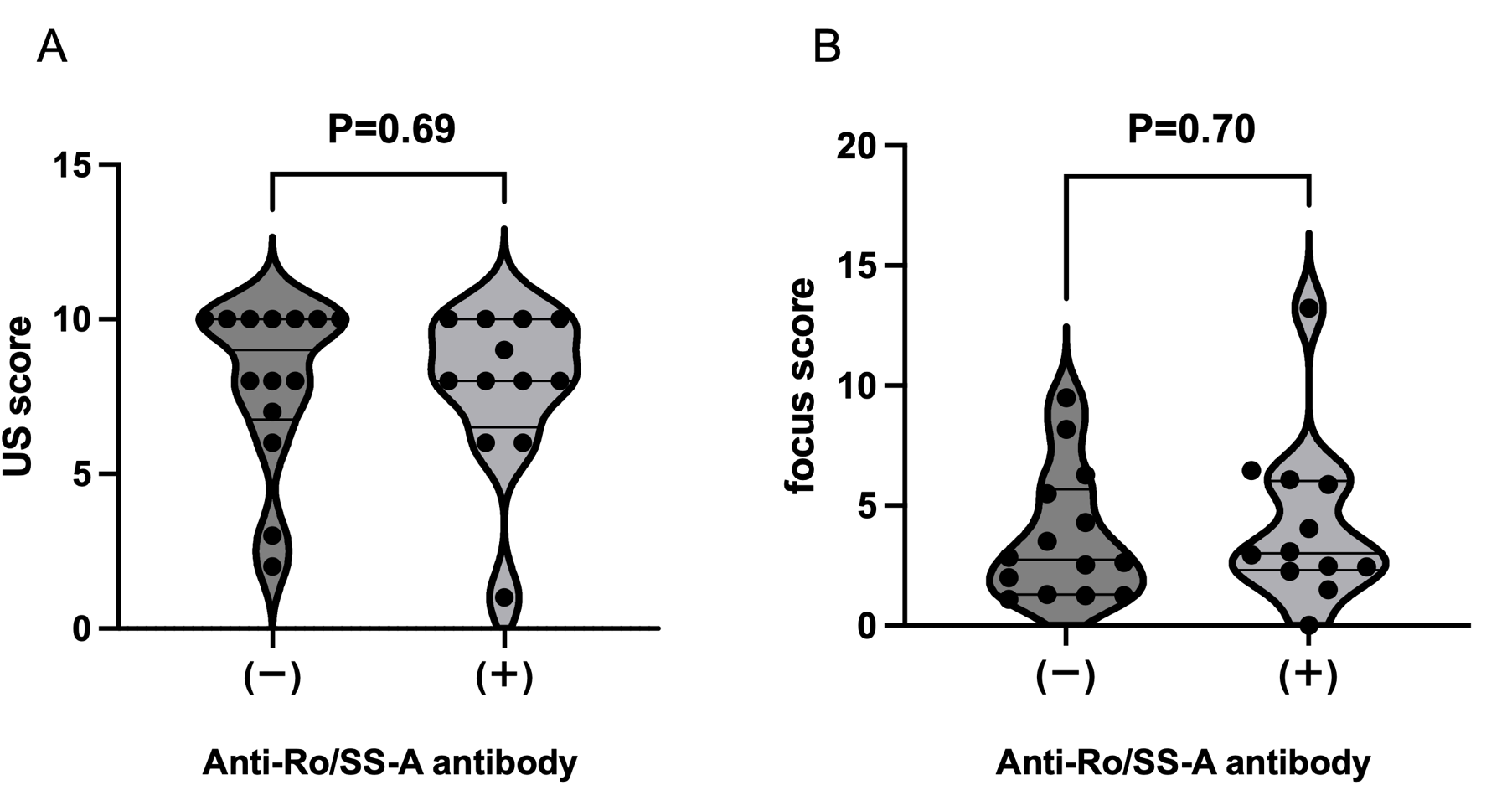
**

**Supplementary Fig. S1. Ultrasonography score and focus score in anti-centromere antibody positive patients with Sjögren's syndrome and anti-Ro/SS-A antibody and those without anti-Ro/SS-A antibody**

The ultrasonography score (A) and focus score (B) between patients with anti-centromere antibody positive Sjögren's syndrome and anti-Ro/SS-A antibody (n = 12) and those without anti-Ro/SS-A antibody (n = 14) were compared using the Mann–Whitney U test. The distributions are presented using violin plots, which included all the data points. Statistical significance was set a t P < 0.05. ACA, anti-centromere antibody; SS, Sjögren's syndrome; US, ultrasonography.
